# Genistein alleviates H_2_O_2_-induced senescence of human umbilical vein endothelial cells via regulating the TXNIP/NLRP3 axis

**DOI:** 10.1080/13880209.2021.1979052

**Published:** 2021-10-18

**Authors:** Guihua Wu, Siming Li, Guangjin Qu, Jiajia Hua, Jing Zong, Xiaofeng Li, Fanghui Xu

**Affiliations:** a Department of Geriatrics, Nantong First Geriatric Hospital, Nantong City, China; b Department of Geriatrics, Harbin Second Hospital, Harbin, China; c Cadre Ward of The First Affiliated Hospital of Harbin Medical University, Harbin City, China; d Department of Traditional Chinese Medicine, Nantong First Elderly Hospital, Nantong City, China; e Department of Otolaryngology, East Hospital, Shanghai Sixth People's Hospital, Nanhui New City, China

**Keywords:** Thioredoxin-interacting protein, nucleotide-binding and oligomerization domain-like receptor 3, cell senescence, inflammasome, cardiovascular disease

## Abstract

**Context:**

Genistein (Gen) has shown protective effects against ageing process.

**Objective:**

To explore the role of Gen on the senescence of H_2_O_2_-induced human umbilical vein endothelial cells (HUVECs) and investigate the possible mechanism.

**Materials and methods:**

HUVECs were treated with different concentrations of H_2_O_2_ (50, 100, 200 and 400 μmol/L) for 1 h or Gen administration (20, 40, 80 and 160 μg/mL) for 24 h. Functional experiments (cell counting kit-8, β-galactosidase staining and flow cytometry) were used to detect the effect of Gen on H_2_O_2_-induced HUVECs. After HUVECs were transfected with TXNIP overexpression plasmids, the expression of p16, p21, thioredoxin-interacting protein (TXNIP), nucleotide-binding and oligomerization domain-like receptor 3 (NLRP3), cleaved caspase-3 and cleaved caspase-1 in HUVECs were detected by quantitative real-time polymerase chain reaction (qRT-PCR) and western blot.

**Results:**

H_2_O_2_ (200 and 400 μmol/L) inhibited the proliferation of HUVECs. At concentrations of >50 μmol/L, H_2_O_2_ induced the cell cycle progression arrests in G1 phase and promoted cell senescence of HUVECs. Gen had no obvious cytotoxicity to HUVECs below 160 µg/mL. H_2_O_2_-induced HUVEC senescence and the expression of TXNIP and NLRP3 in HUVECs were down-regulated by Gen (40 and 80 µg/mL). Expressions of TXNIP and NLRP3 in HUVECs were up-regulated by H_2_O_2_ but down-regulated by Gen. Overexpressed TXNIP partially reversed the suppressive effect of Gen on H_2_O_2_-induced senescence and apoptosis of HUVECs. Expressions of p16, p21, TXNIP, NLRP3, cleaved caspase-3 and cleaved caspase-1 in H_2_O_2_-treated HUVECs were inhibited by Gen, while the inhibition as such was partially reversed by overexpressed TXNIP.

**Discussion and conclusions:**

H_2_O_2_-induced HUVEC senescence was alleviated by Gen via suppressing the TXNIP/NLRP3 axis, which may offer a potential therapeutic approach for improving HUVEC senescence and provide a new direction for the treatment of cardiovascular disease.

## Introduction

Cardiovascular diseases are usually associated with ageing, with rapidly increasing incidence in recent decades (Du et al. 2019). Emerging evidence has implied that vascular diseases are closely related to the dysfunction of vascular endothelial cells (Hafner et al. 2014; Sikora et al. 2014). Loss of replication ability of senescent endothelial cells destroys cell integrity and inhibits angiogenesis (Cardus et al. 2013). The interrelation between ageing and endothelial dysfunction reveals that discovering novel ways to resist endothelial senescence is vital (Song et al. 2014).

Hydrogen peroxide (H_2_O_2_) is a stressor that can induce senescence, and the induced senescence process could imitate the similar conditions occurring in ageing population with high efficiency (Toussaint et al. 2000). In line with the published literature, human umbilical vein endothelial cells (HUVECs) could increase β-galactosidase positive cells by H_2_O_2_ in a dose-dependent manner (Lin et al. 2011).

Genistein (Gen; 4′,5,7-trihydroxyisoflavone) is an isoflavone extracted from soy products with similar structure to oestrogen (Mansour et al. 2017) and is widely employed as an antioxidant and anti-inflammatory agent (Ibrahim et al. 2016). Gen has been demonstrated to ameliorate endothelial nitric oxidase synthase uncoupling in oxidized low-density lipoprotein-induced HUVECs by up-regulating the sirtuin-1 pathway (Zhang et al. 2016). However, the effects of Gen on H_2_O_2_-induced HUVEC senescence have not been clearly clarified.

The nucleotide-binding and oligomerization domain-like receptor 3 (NLRP3) inflammasome, identified as a pattern recognition receptor, consists of NLRP3, apoptosis-associated speck-like protein (ASC) and procaspase-1 (Schroder and Tschopp 2010). Thioredoxin (TRX) is expressed in almost all species and functions as an essential role in modulating cellular redox status (Lu and Holmgren 2014). Thioredoxin-interacting protein (TXNIP), also known as thioredoxin binding protein-2, interacts and negatively regulates the expression and function of TRX, thereby participating in the cellular redox balance (Alhawiti et al. 2017). Further studies showed that deficiency of TXNIP impairs the activation of the NLRP3 inflammasome and subsequent secretion of interleukin-1 beta (IL-1β), which may have a powerful impact on inflammatory response (Cao et al. 2017). For instance, hepatic expression of TXNIP and the interaction between TXNIP and NLRP3 were promoted in the acute liver failure mouse model, indicating that TXNIP-mediated activation of NLRP3 inflammasome is essential for acute liver failure (Liu et al. 2016). Importantly, TXNIP–NLRP3 inflammasome is activated by trimethylamine-N-oxide which finally contributes to inflammation and endothelial dysfunction in HUVECs according to the previous study (Sun et al. 2016). Nevertheless, few studies have been performed on the role of TXNIP–NLRP3 inflammasome in H_2_O_2_-induced HUVEC senescence.

This paper assesses the effect of Gen on H_2_O_2_-induced HUVEC senescence and identify whether TXNIP–NLRP3 inflammasome was involved in the senescence process.

## Materials and methods

### Cell culture, transfection

Human umbilical vein endothelial cells were obtained from ScienCell Research Laboratories (#8000, Carlsbad, CA) and cultured in Medium 199 (12350039, Gibco™, Thermo Fisher Scientific, Waltham, MA) which contained 50 U/mL penicillin-streptomycin (P1400, Solarbio, Beijing, China) and 50 mL of endothelial cell growth supplement (E0760, Sigma-Aldrich, St. Louis, MO). The medium was then added with 2.2 g/L sodium bicarbonate (YZ-1613655, Solarbio, Beijing, China) and 10% foetal bovine serum (FBS; SH30070, Hyclone, Logan, UT). The cells were incubated in a humidified atmosphere at 37 °C with 5% CO_2_.

The pcDNA3.1 plasmid (VT1010, YouBio, Xi'an, China) carrying the TXNIP gene was transfected into HUVECs (overexpressed TXNIP group; TXNIP) to perform TXNIP overexpression using Lipofectamine 2000 reagent (Invitrogen, Carlsbad, CA) in accordance with the kit instructions, accompanied by empty plasmid (empty plasmid group; NC) as the control. Briefly, 2.0 × 10^5^ HUVECs were seeded in the 24-well plate. First, 0.8 μg DNA and 2.0 µL liposome were diluted in 50 µL serum free basic medium (Gibco, Carlsbad, CA), and then stood at room temperature for 5 min. After that, the diluted DNA was mixed with the diluted liposome and incubated for 20 min at room temperature to form 100 µL complexes which was then added into cells and co-cultured at 37 °C for 48 h.

For silencing TXNIP, small interfering RNA (siTXNIP, 5′-AAGCCGTTAGGATCCTGGCTT-3′) and siNC (5′-AATTCTCCGAACGTGTCACGT-3′) were purchased from GenePharma (Shanghai, China), where siNC was used as the negative control (NC). HUVECs were transfected with 50 nM siTXNIP or siNC using Lipofectamine 2000 reagent (Invitrogen, Carlsbad, CA) as per the kit instructions.

### Grouping

In order to detect the effects of Gen (PHR1859, Sigma-Aldrich, St. Louis, MO) on H_2_O_2_-induced HUVEC senescence, the experimental grouping was designed as follows: 200 μmol/L H_2_O_2_ group (H_2_O_2_), 200 μmol/L H_2_O_2_+40 μg/mL Gen group (H_2_O_2_+Gen40), 200 μmol/L H_2_O_2_+80 μg/mL Gen group (H_2_O_2_+Gen80), 40 μg/mL Gen group (Gen40) and 80 μg/mL Gen group (Gen80). The cells were pre-treated with Gen for 24 h and then exposed to H_2_O_2_ for 1 h, both at 37 °C.

To investigate the roles of overexpressed TNXIP and Gen in H_2_O_2_-induced senescence and apoptosis of HUVECs, the experimental grouping was designed as follows: NC group, overexpressed TXNIP group (TXNIP), NC + 200 μmol/L H_2_O_2_ group (NC + H_2_O_2_), NC + 80 μg/mL Gen group (NC + Gen80), NC + 200 μmol/L H_2_O_2_+80 μg/mL Gen group (NC + H_2_O_2_+Gen80), overexpressed TXNIP + 200 μmol/L H_2_O_2_ group (TXNIP + H_2_O_2_), overexpressed TXNIP + 80 μg/mL Gen group (TXNIP + Gen80) and overexpressed TXNIP + 200 μmol/L H_2_O_2_+80 μg/mL Gen group (TXNIP + H_2_O_2_+Gen80). The cells were transfected with overexpressed TNXIP plasmid or NC, pre-treated with Gen for 24 h and then exposed to H_2_O_2_ for 1 h, both at 37 °C.

To investigate the roles of silencing TNXIP and Gen in H_2_O_2_-induced senescence of HUVECs, the experimental grouping was designed as follows: siNC, siTXNIP, siNC + H_2_O_2_, siNC + Gen80, siNC + H_2_O_2_+Gen80, siTXNIP + H_2_O_2_, siTXNIP + Gen80 and siTXNIP + H_2_O_2_+Gen80 group. The cells were transfected with siTXNIP or siNC, pre-treated with Gen for 24 h and then exposed to H_2_O_2_ for 1 h, both at 37 °C.

### CCK-8 assay

After treatment with different concentrations of H_2_O_2_ or Gen administration, HUVEC viability was measured by a Cell Counting Kit-8 (HY-K0301, MedChemExpress, South Brunswick Township, NJ) on the basis of the manufacturer’s instructions. In brief, the treated HUVEC suspension was inoculated in 96-well plates (100 μL/well). Next, the plates were incubated in a humidified incubator at 37 °C with 5% CO_2_. Then the cells were supplemented with 10 μL of the CCK-8 solution and incubated for 3 h. Finally, the absorbance at 450 nm was measured using a microplate reader (TriStar^2^ LB 942, Berthold Technologies GmbH & Co. KG, Bad Wildbad, Germany).

### HUVEC senescence detection

A β-galactosidase Staining Kit (G1580, Solarbio, Beijing, China) was employed to investigate the senescence status of HUVECs after the cells were treated with 0, 20, 40, 80 and 160 μg/mL Gen. Specifically, the third passage of HUVECs (100 cells) at logarithmic growth stage was seeded in a six-well plate, and then the medium was removed and washed with phosphate-buffered saline (PBS; D8537, Sigma-Aldrich, St. Louis, MO) once. Next, 1 mL of β-galactosidase staining stationary liquid was added, and the cells were then incubated for 15 min at room temperature. After incubation, the stationary liquid was removed and the cells were washed with PBS for three times (3 min/time). Subsequently, PBS was removed and 1 mL of β-galactosidase staining working solution was added into the cells strictly following the kit instructions. The cells were then incubated overnight at 37 °C. The β-galactosidase positive cells were calculated using ImageJ software (Version 1.52v, National Institutes of Health, Bethesda, MD) while its status was observed under a microscope (BX53M, OLYMPUS, Tokyo, Japan).

### Detection of HUVEC apoptosis and cell cycle distribution

Cell cycle distribution was investigated via a Cell Cycle and Apoptosis Analysis Kit (C1052, Beyotime, Shanghai, China). The HUVECs were collected, washed with 1 mL of cold PBS and fixed with 70% pre-cold ethanol for 24 h. Subsequently, the cells were centrifuged at 1000×*g* for 5 min and pelleted. Next, the supernatant was discarded and the cells were washed with 1 mL of cold PBS. Afterwards, each sample was resuspended in 0.5 mL of PBS with 25 µL of PI (20×) and 10 µL of RNase A (50×). The cells were then incubated at 37 °C for 30 min in the dark. Cell cycle analysis was performed using the Invitrogen Attune flow cytometer (Thermo Fisher Scientific, Waltham, MA), and the percentage of HUVECs in G0/G1, S and G2/M phases was calculated by ModFit LT analysis program (Verity Software House, Topsham, ME).

After H_2_O_2_ or Gen treatment, or 48-h transfection, HUVEC apoptosis was detected using the ANNEXIN V-FITC/PI kit (CA1020, Solarbio, Beijing, China) under the manufacturer’s instructions. Specifically, 27 mL of deionized water was added into 3 mL of binding buffer (10×). The HUVECs were collected after trypsinization, and then washed with cold PBS. Next, the cells were suspended in 1 mL of 1× binding buffer, centrifuged at 300×*g* for 10 min and then the supernatant was removed. Subsequently, the cells were re-suspended in 1 mL of 1× binding buffer in order to adjust the cell density to 1 × 10^6^/mL. After that, the cells (100 μL) were supplemented with 5 μL of Annexin V-FITC for 10-min incubation and were then added with 5 μL of PI for 5-min incubation at room temperature in the dark. The staining was identified using the ACEA NovoCyte flow cytometer (ACEA Biosciences, San Diego, CA) at a wavelength of 490 nm.

### Determination of ROS

ROS level was estimated using 2′,7′-dichlorofluorescin diacetate (DCFH-DA, Sigma, St. Louis, MO). HUVECs were detached with trypsin–EDTA, collected by centrifugation, and washed with PBS. The cells were treated with 10 µM DCFH-DA for 30 min at 37 °C. Fluorescent intensity was analysed using a flow cytometer (BD Biosciences, Franklin Lakes, NJ).

### Western blot

For immunoblotting, total proteins were collected using RIPA buffer (R0278, Sigma-Aldrich, St. Louis, MO). Protein concentration was determined using the BCA method (Beyotime, Shanghai, China). The extracted proteins were separated by 10% sodium dodecyl sulphate polyacrylamide gel electrophoresis (SDS-PAGE) and then transferred to polyvinylidene difluoride (PVDF) membranes. The membranes were blocked in TBS-Tw (20 mM Tris pH 7.4, 150 mM NaCl, 0.2% Tween-20) with 5% skimmed milk for 60 min and then incubated overnight at 4 °C with the following primary antibodies: anti-TXNIP antibody (rabbit, ab188865, 1:1000, Abcam, Cambridge, UK), anti-NLRP3 antibody (rabbit, ab214185, 1:1000, Abcam, Cambridge, UK), anti-p16 antibody (mouse, ab201980, 1:1000, Abcam, Cambridge, UK), anti-p21 antibody (rabbit, ab109199, 1:1000, Abcam, Cambridge, UK), anti-cleaved caspase-3 antibody (rabbit, ab2302, 1:1000, Abcam, Cambridge, UK), anti-caspase-3 antibody (rabbit, ab32351, 1:5000, Abcam, Cambridge, UK), anti-cleaved caspase-1 antibody (rabbit, #4199, 1:1000, Cell Signaling Technology, Boston, MA), anti-caspase-1 antibody (rabbit, #3866, 1:1000, Cell Signaling Technology, Boston, MA) and anti-GAPDH antibody (mouse, ab8245, 1:1000, Abcam, Cambridge, UK). GAPDH was employed as the normalization reference. The membranes were then incubated with the secondary horseradish peroxidase (HRP)-conjugated antibodies goat anti-rabbit IgG H&L (HRP) (goat, ab205718, 1:2000, Abcam, Cambridge, UK) and goat anti-mouse IgG H&L (HRP) (goat, ab205719, 1:2000, Abcam, Cambridge, UK) for 1 h at room temperature. Afterwards, protein bands were visualized using an enhanced chemiluminescence (ECL) kit (ab65623, Abcam, Cambridge, UK) and their grey values were gathered and calculated using ImageJ software (Version 1.52v, National Institutes of Health, Bethesda, MD) (Chen et al. 2018).

### Quantitative real-time polymerase chain reaction (qRT-PCR)

Total RNA isolation and cDNA synthesis were carried out in line with commercial standard methods. Briefly, total RNA was extracted from the HUVECs with TRIzol reagent (93289, Sigma-Aldrich, St. Louis, MO). A total of 2 μL cDNA was synthesized with the iScript cDNA synthesis kit (Bio-Rad Laboratories, Inc., Hercules, CA). RT-qPCR was then performed using Syber^®^ Premix Ex TaqTMII (RR820L, Takara, Otsu, Japan) in the 7500 Real-Time PCR System (Applied Biosystems, Foster City, CA). The primer sequences used for real-time PCR are listed in Table 1, and the PCR cycle program was set as follows: initial denaturation at 95 °C for 10 s, 40 cycles of denaturation at 95 °C for 5 s and annealing at 60 °C for 20 s. Relative gene expression was normalized to GAPDH and calculated by the 2^−ΔΔCt^ method (Livak and Schmittgen 2001).

**Table 1.** Primers for qRT-PCR.

Gene	Primers
*TXNIP*	
Forward	5′-CCCTGGTAATTGGCAGCAGA-3′
Reverse	5′-TGCAGGGATCCACCTCAGTA-3′
*NLRP3*	
Forward	5′-CGTTCCAGGGAGTCGTTTGA-3′
Reverse	5′-TTTGCACGAAGTCCTCCTCC-3′
*GAPDH*	
Forward	5′-GATTTGGTCGTATTGGGCGC-3′
Reverse	5′-TTCCCGTTCTCAGCCTTGAC-3′
*p16*	
Forward	5′-ATGATGATGGGCAGCGCC-3′
Reverse	5′-CGAGGTTTCTCAGAGCCT-3′
*p21*	
Forward	5′-CTGCCCAAGCTCTACCTTCC-3′
Reverse	5′-TCGACCCTGAGAGTCTCCAG-3′

### Statistical analysis

Each experiment was performed for three times. Results were presented as mean ± standard deviation (SD). Data multiple comparisons were analysed by one-way ANOVA using SPSS version 22.0 (SPSS Inc., Chicago, IL). *p* < 0.05 was considered as statistically significant.

## Results

### Assessment of H_2_O_2_-induced HUVEC senescence

HUVECs have now become the main *in vitro* experimental materials for vascular endothelial cell related research. Compared with other sources of vascular endothelial cells, HUVECs are derived from the umbilical cord of newborns, which resemble human arterial endothelial cells in morphology and physiological functions. It is more convincing when the experimental results conform to the real situation of the human body. In addition, HUVECs are easy to culture with strong proliferation and division abilities as well as short passage time, thus saving experimental time.

**Figure 1.**
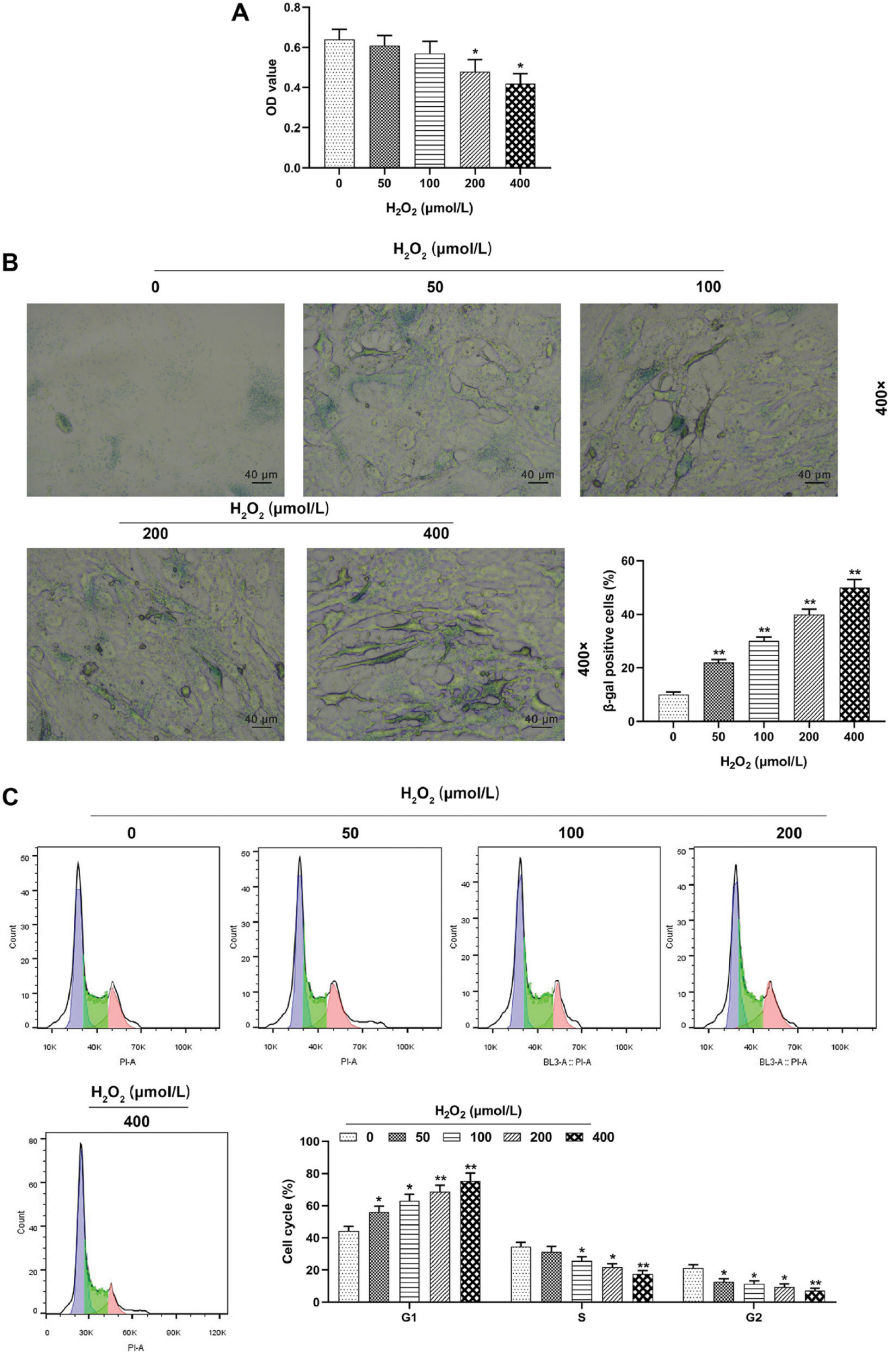
Proliferation and cell cycle G1/S transition of HUVECs were inhibited but cell senescence was promoted by H_2_O_2_. (A) HUVEC viability detection was executed by CCK-8 assay after separate treatment with 0, 50, 100, 200 and 400 μmol/L H_2_O_2_ for 24 h. (B) HUVECs senescence was investigated by β-galactosidase staining after separate treatment with 0, 50, 100, 200 and 400 μmol/L H_2_O_2_ for 24 h. Magnification: ×400, scale bar = 40 µm. (C) Cell cycle progression of HUVECs was identified by flow cytometry after separate treatment with 0, 50, 100, 200 and 400 μmol/L H_2_O_2_ for 24 h. All experiments were performed in triplicate and the experimental data were expressed as mean ± standard deviation (SD) (*p < 0.05, **p < 0.01, vs. 0 μmol/L H_2_O_2_). HUVECs: human umbilical vein endothelial cells; CCK-8: Cell Counting Kit-8.

In this phase, HUVECs were separately treated with 0, 50, 100, 200 and 400 μmol/L H_2_O_2_ for 24 h, to uncover the role of different concentrations of H_2_O_2_ on the viability, senescence and cell cycle progression of HUVECs. In line with the results of CCK-8 assay exhibited in Figure 1(A), HUVEC viability showed a downward trend after H_2_O_2_ treatment, and specifically, as the concentration of H_2_O_2_ increased, its ability to inhibit cell activity became stronger (*p* < 0.05). Meanwhile, Figure 1(B) clearly depicted that after β-galactosidase staining, the percentage of dark blue cells, as β-galactosidase positive cells, was up-regulated with the increase of H_2_O_2_ concentration, suggesting that HUVEC senescence was promoted by H_2_O_2_ and the promoting effect was enhanced as H_2_O_2_ concentration increased (*p* < 0.01). Additionally, the cell cycle progression of HUVECs treated with different concentrations of H_2_O_2_ was identified by flow cytometry. Figure 1(C) reveals that the percentage of HUVECs was up-regulated in G1 phase but down-regulated in S and G2 phases with the increase of H_2_O_2_ concentration, indicating that the cell cycle G1/S transition of HUVECs was inhibited by H_2_O_2_ in a dose-dependent manner (*p* < 0.05).

### Effect of Gen on HUVECs proliferation, senescence and cell cycle progression

**Figure 2.**
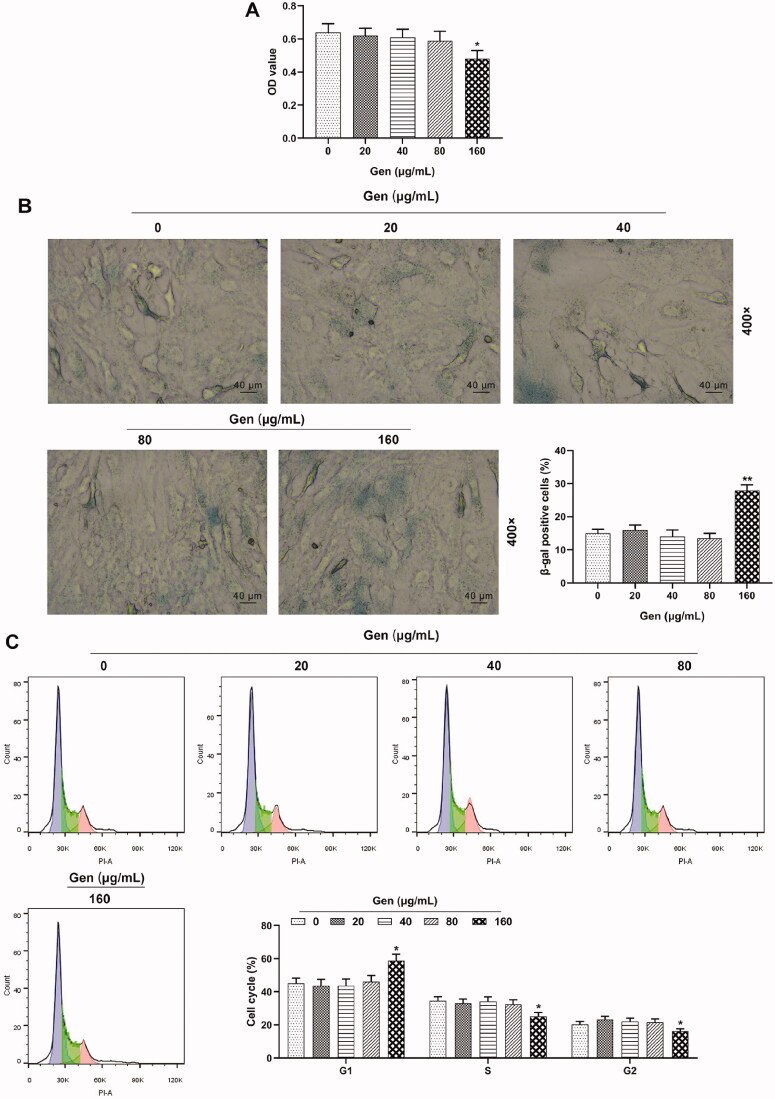
Gen below 160 µg/mL had no obvious cytotoxicity on HUVECs. (A) HUVEC viability was detected by CCK-8 assay after separate treatment with 0, 20, 40, 80 and 160 μg/mL Gen (Genistein) for 24 h. (B) HUVEC senescence was investigated by β-galactosidase staining after separate treatment with 0, 20, 40, 80 and 160 μg/mL Gen for 24 h. Magnification: ×400, scale bar = 40 µm. (C) Cell cycle progression of HUVECs was identified by flow cytometry after separate treatment with 0, 20, 40, 80 and 160 μg/mL Gen for 24 h. All experiments were performed in triplicate and the experimental data were expressed as mean ± standard deviation (SD) (*p < 0.05, **p < 0.01, vs. 0 μg/mL Gen). HUVECs: human umbilical vein endothelial cells; CCK-8: Cell Counting Kit-8.

In order to detect the possible cytotoxicity of Gen on the viability, senescence and cell cycle progression of HUVECs, the HUVECs were separately treated with 0, 20, 40, 80 and 160 μg/mL Gen for 24 h. The results of CCK-8 demonstrated that compared with 0 μg/mL Gen, HUVEC viability showed no significant difference after treatment with 20, 40 and 80 μg/mL Gen, but the viability was decreased after treatment with 160 μg/mL Gen (Figure 2(A), *p* < 0.05). As shown in Figure 2(B), the results of β-galactosidase staining demonstrated that in contrast with 0 μg/mL Gen, the percentage of β-galactosidase positive cells was not obviously increased after the cells were treated with 20, 40 and 80 μg/mL Gen, while it was up-regulated after treatment with 160 μg/mL Gen (*p* < 0.01). Moreover, according to the results of flow cytometry in Figure 2(C), taking 0 μg/mL Gen as the comparison, the percentage of HUVECs treated with 20, 40 and 80 μg/mL Gen showed no evident changes in G1, S and G2 phases, but that of HUVECs treated with 160 μg/mL Gen was increased in G1 phases and decreased in S and G2 phases (*p* < 0.05). Therefore, to eliminate the cytotoxicity of Gen on cells, 40 and 80 μg/mL Gen were used in the subsequent tests.

### Gen had a protective effect on H_2_O_2_-induced HUVEC senescence

**Figure 3.**
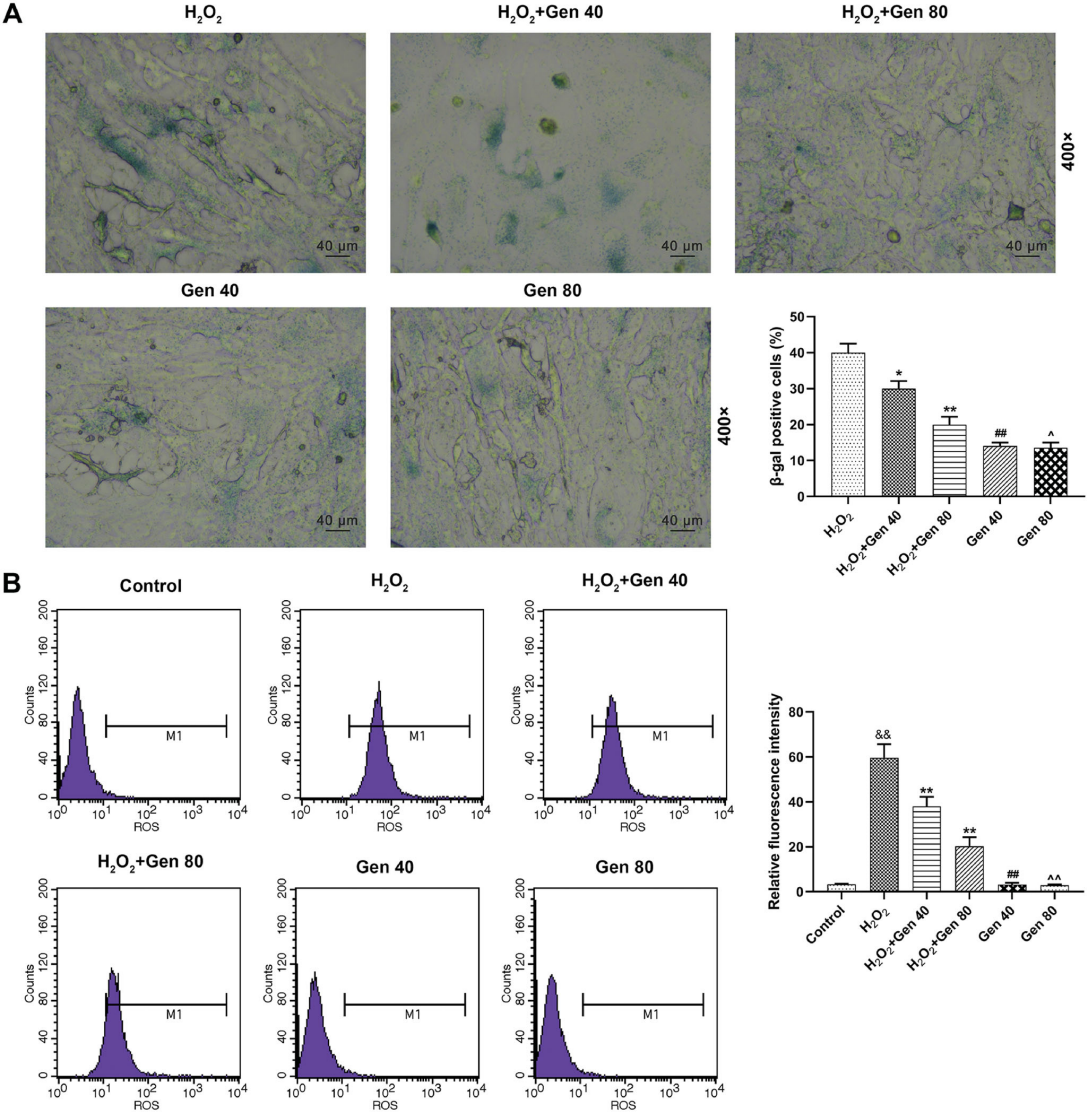
H_2_O_2_-induced HUVEC senescence and ROS level were reversed by Gen. (A) H_2_O_2_-induced HUVEC senescence after 40 and 80 μg/mL Gen treatment for 24 h was detected by β-galactosidase staining. Magnification: ×400, scale bar = 40 µm. (B) The level of ROS H_2_O_2_-induced HUVEC after 40 and 80 μg/mL Gen treatment for 24 h was detected by flow cytometry. All experiments were performed in triplicate and the experimental data were expressed as mean ± standard deviation (SD) (^&&^*p*< 0.01, vs. control; *p< 0.05, **p< 0.01, vs. H_2_O_2_; ^##^*p*< 0.01, vs. H_2_O_2_+Gen40; ^∧^*p*< 0.05, ^∧∧^*p*< 0.01, vs. H_2_O_2_+Gen80) HUVECs: human umbilical vein endothelial cells.

Based on the data of the previous experiment, 40 and 80 μg/mL Gen were employed in the following experiments to unveil its effect on H_2_O_2_-induced HUVEC senescence, TXNIP/NLRP3 axis. The results of β-galactosidase staining shown in Figure 3(A) revealed that the percentage of β-galactosidase positive cells in H_2_O_2_+Gen40 and H_2_O_2_+Gen80 groups was lower than in H_2_O_2_ group (*p* < 0.05), and moreover, the decreased percentage of β-galactosidase positive cells in Gen40 group and Gen80 group was shown as compared with H_2_O_2_+Gen40 group (*p* < 0.01) and H_2_O_2_+Gen80 group (*p* < 0.05), respectively. These discoveries demonstrated that H_2_O_2_-induced HUVEC senescence was alleviated by Gen.

In addition, the effect of Gen on the oxidative stress induced by H_2_O_2_ was detected. As shown in Figure 3(B), the ROS level was significantly increased in H_2_O_2_ group (*p* < 0.01), and that in H_2_O_2_+Gen40 and H_2_O_2_+Gen80 groups was lower than in H_2_O_2_ group (*p* < 0.01). The ROS level was down-regulated in Gen40 group in comparison with that in H_2_O_2_+Gen40 group (*p* < 0.01), and that was also down-regulated in Gen80 group as compared with H_2_O_2_+Gen80 group (*p* < 0.05).

**Figure 4.**
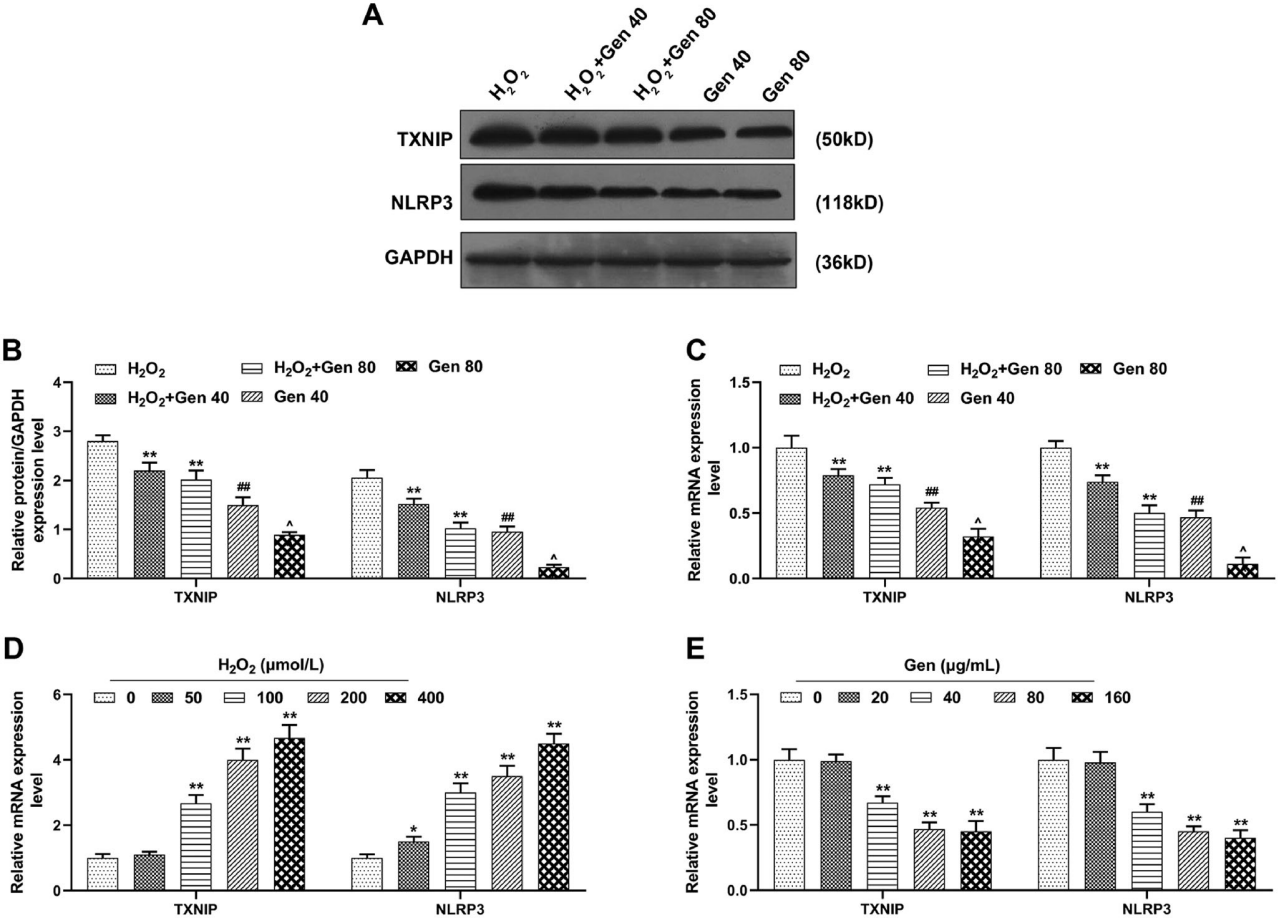
The expression of TXNIP and NLRP3 of H_2_O_2_-induced HUVEC was reversed by Gen. (A–C) Effects of 40 and 80 μg/mL Gen on the expression of TXNIP and NLRP3 in HUVECs after 200 μmol/L H_2_O_2_ treatment were investigated by quantitative real-time polymerase chain reaction (qRT-PCR) and western blot. GAPDH was used as the internal control. (D) Effects of different concentrations of H_2_O_2_ on the expression of TXNIP and NLRP3 in HUVECs was determined by qRT-PCR. GAPDH was used as the internal control. (E) Effects of different concentrations of Gen on the expression of TXNIP and NLRP3 in HUVECs was determined by qRT-PCR. GAPDH was used as the internal control. All experiments were performed in triplicate and the experimental data were expressed as mean ± standard deviation (SD) (*p< 0.05, **p< 0.01, vs. H_2_O_2_ or 0 μmol/L H_2_O_2_ or 0 μg/mL Gen; ^##^*p*< 0.01, vs. H_2_O_2_+Gen40; ^∧^*p*< 0.05, vs. H_2_O_2_+Gen80). HUVECs: human umbilical vein endothelial cells; TXNIP: thioredoxin-interacting protein; NLRP3: nucleotide-binding and oligomerization domain-like receptor 3.

In order to measure the expression of TXNIP and NLRP3 in HUVECs, qRT-PCR and western blot was employed. As exhibited in Figure 4(A–C), the expression of TXNIP and NLRP3 in H_2_O_2_+Gen40 and H_2_O_2_+Gen80 groups was lower than those in H_2_O_2_ group (*p* < 0.01). Their expression was down-regulated in Gen40 group in comparison with those in H_2_O_2_+Gen40 group (*p* < 0.01), and those were also down-regulated in Gen80 group as compared with H_2_O_2_+Gen80 group (*p* < 0.05). These findings revealed that the expression of TXNIP and NLRP3 in HUVECs after H_2_O_2_ treatment was down-regulated by Gen.

### Expressions of TXNIP and NLRP3 in HUVECs was regulated by H_2_O_2_ and Gen

To uncover the role of different concentrations of H_2_O_2_ or Gen in the expression of TXNIP and NLRP3 in HUVECs, the HUVECs were separately treated with 0, 50, 100, 200 and 400 μmol/L H_2_O_2_ or 0, 20, 40, 80 and 160 μg/mL Gen for 24 h. It can be concluded from the results of qRT-PCR that the relative mRNA expression of TXNIP and NLRP3 in HUVECs was promoted by H_2_O_2_ and the promoting effect was enhanced with the increase of H_2_O_2_ concentration (Figure 4(D), *p* < 0.05). By contrast, those in HUVECs were reduced by Gen and the suppressive effect was enhanced with the increase of Gen concentration (Figure 4(E), *p* < 0.01).

### Overexpressed TXNIP partially reversed the effect of Gen on H_2_O_2_-induced senescence and apoptosis of HUVECs

**Figure 5.**
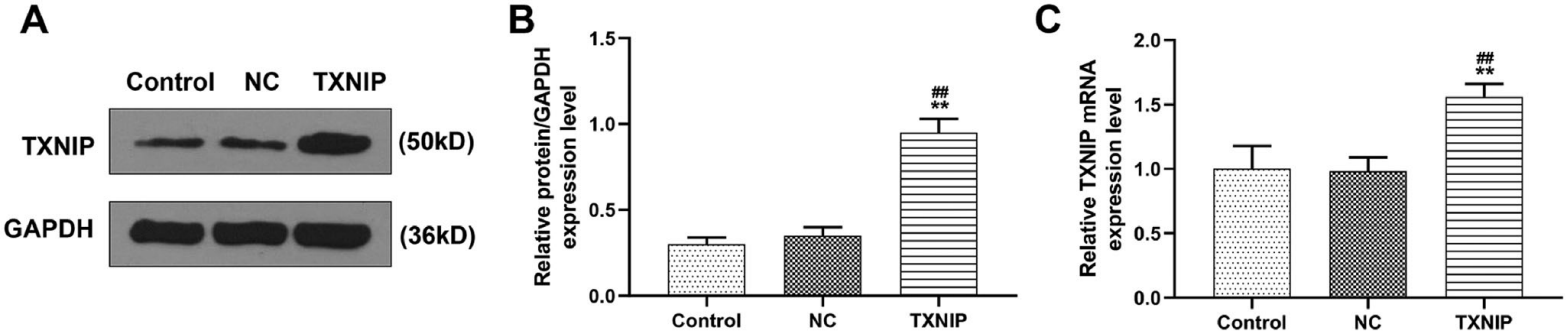
Overexpressed TXNIP plasmid was successfully transfected into HUVECs. (A, B) The transfection efficiency was determined by western blot. (C) The transfection efficiency was determined by qRT-PCR. GAPDH was used as the internal control. All experiments were performed in triplicate and the experimental data were expressed as mean ± standard deviation (SD) (**p< 0.01, vs. control; ^##^*p*< 0.01, vs. NC). HUVECs: human umbilical vein endothelial cells; TXNIP: thioredoxin-interacting protein; NC: negative control.

In this phase, overexpressed TXNIP plasmid was transfected into HUVECs to further unveil the effect of overexpressed TXNIP on H_2_O_2_-induced senescence and apoptosis of HUVECs, and to discover whether the effects of Gen on HUVECs could be influenced by overexpressed TXNIP. The mRNA and protein expression of TXNIP in TXNIP group was higher than those in control and NC groups (Figure 5(A–C), *p* < 0.01).

**Figure 6.**
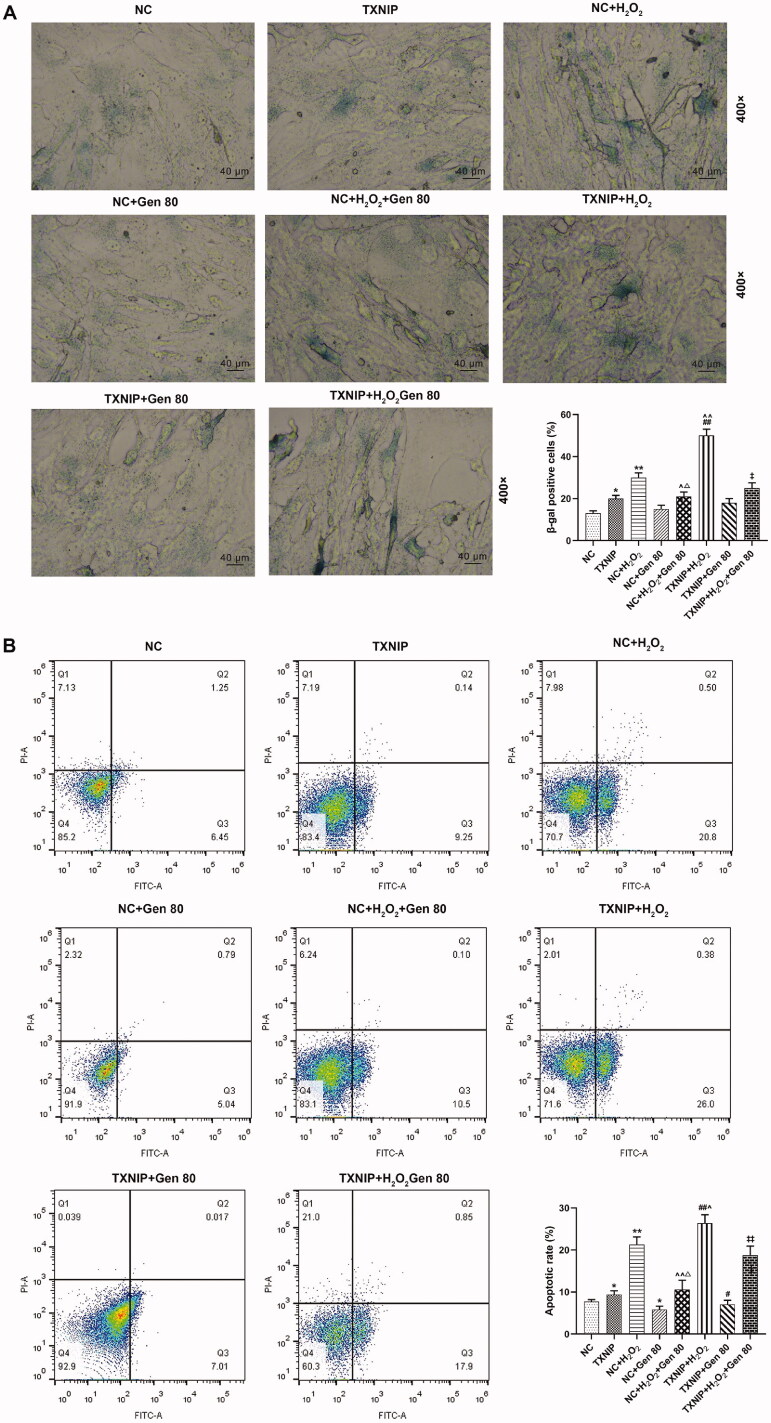
Overexpressed TXNIP partially reversed the effect of Gen on H_2_O_2_-induced senescence and apoptosis of HUVECs. HUVECs were transfected with overexpressed TXNIP plasmid, treated with H_2_O_2_ or Gen alone or combination. (A) After transfection of overexpressed TXNIP plasmid, H_2_O_2_-induced HUVEC senescence after 80 μg/mL Gen treatment was detected by β-galactosidase staining. Magnification: ×400, scale bar = 40 µm. (B) After transfection of overexpressed TXNIP plasmid, H_2_O_2_-induced HUVEC apoptosis after 80 μg/mL Gen treatment was detected by flow cytometry. All experiments were performed in triplicate and the experimental data were expressed as mean ± standard deviation (SD) (*p< 0.05, **p< 0.01, vs. NC; ^∧^*p*< 0.05, ^∧∧^*p*< 0.01, vs. NC + H_2_O_2_; ^#^*p*< 0.05, ^##^*p*< 0.01, vs. TXNIP; ^‡^*p*< 0.05, vs. NC + H_2_O_2_+Gen80; ^Δ^*p*< 0.05, vs. NC + Gen80). HUVECs: human umbilical vein endothelial cells; TXNIP: thioredoxin-interacting protein; NC: negative control.

As shown in Figure 6(A), the results of β-galactosidase staining identified that the percentage of β-galactosidase positive cells in TXNIP and NC + H_2_O_2_ groups was higher than that in NC group (*p* < 0.05). Besides, the percentage of β-galactosidase positive cells in TXNIP + H_2_O_2_ group was up-regulated (*p* < 0.05), but was down-regulated in NC + H_2_O_2_+Gen80 group when compared to those in NC + H_2_O_2_ group (*p* < 0.05). The percentage of β-galactosidase positive cells was increased in TXNIP + H_2_O_2_+Gen80 group compared with that in NC + H_2_O_2_+Gen80 group, so was in NC + H_2_O_2_+Gen80 group compared with that in NC + Gen80 group (Figure 6(A), *p* < 0.05). Moreover, the results of flow cytometry shown in Figure 6(B) demonstrated that the apoptosis rate of HUVECs in TXNIP and NC + H_2_O_2_ group was higher than that in NC group, but that in NC + Gen80 group was lower than in NC group (*p* < 0.05). In addition, the apoptosis rate of HUVECs was increased in TXNIP + H_2_O_2_ group, but decreased in NC + H_2_O_2_+Gen80 group in comparison with NC + H_2_O_2_ group (Figure 6(B), *p* < 0.05). However, the apoptosis rate was enhanced in TXNIP + H_2_O_2_+Gen80 group in comparison with that in NC + H_2_O_2_+Gen80 group (Figure 6(B), *p* < 0.05). The data above unveiled that the suppressive effect of Gen on H_2_O_2_-induced HUVEC senescence and apoptosis was partially reversed by overexpressed TXNIP.

### Overexpressed TXNIP partially reversed the effect of Gen on the expression of senescence genes and senescence-related proteins in H_2_O_2_-treated HUVECs

**Figure 7.**
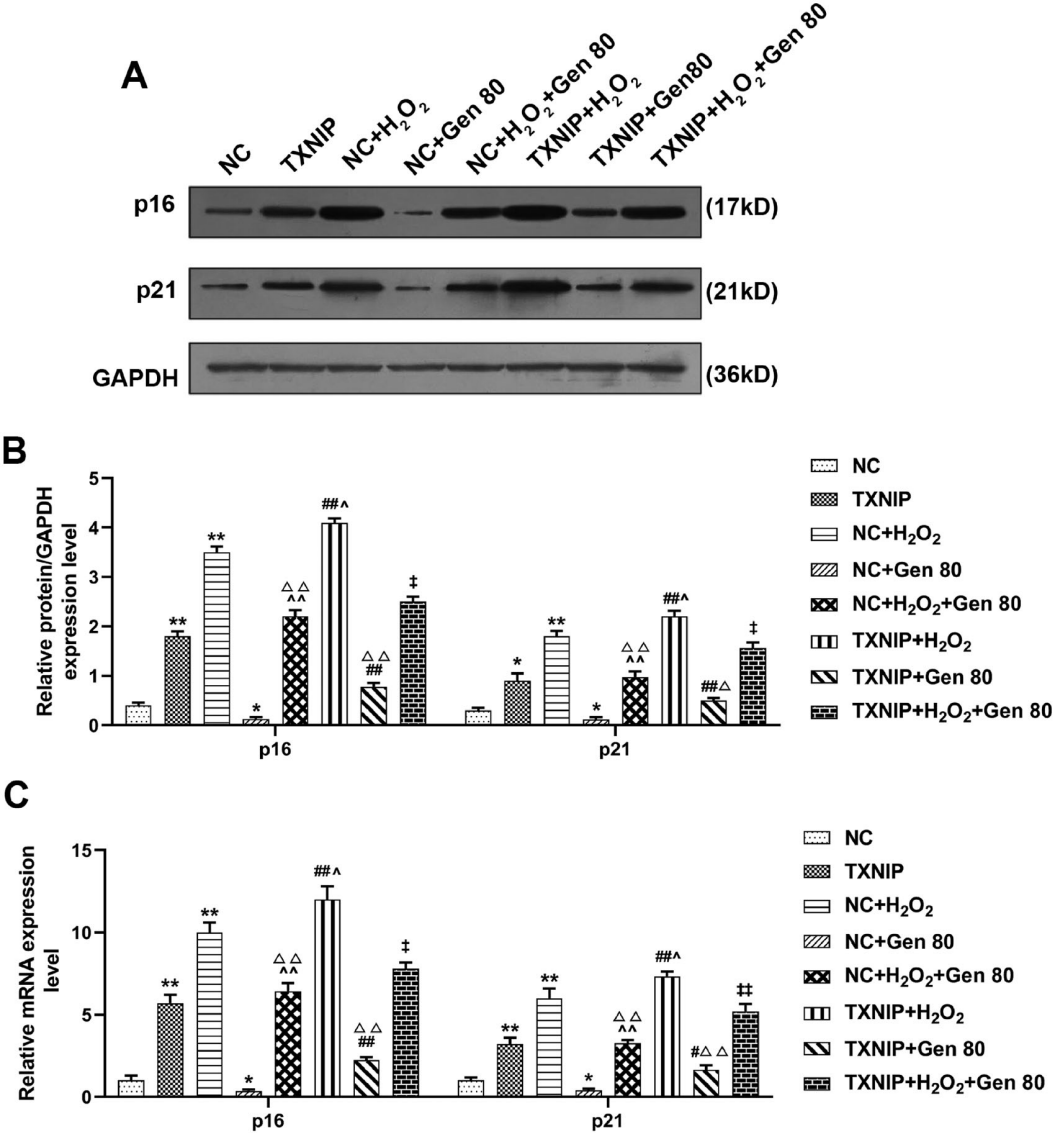
Overexpressed TXNIP partially reversed the effect of Gen on the expression of senescence genes in H_2_O_2_-treated HUVECs. HUVECs were transfected with overexpressed TXNIP plasmid, treated with H_2_O_2_ or Gen alone or combination. (A, B) After transfection of overexpressed TXNIP plasmid, the relative protein expression of p16 and p21 in H_2_O_2_-treated HUVECs under 80 μg/mL Gen treatment were measured by western blot. GAPDH was used as the internal control. (C) After transfection of overexpressed TXNIP plasmid, the relative mRNA expression of p16 and p21 in H_2_O_2_-treated HUVECs under 80 μg/mL Gen treatment were determined by qRT-PCR. GAPDH was used as the internal control. All experiments were performed in triplicate and the experimental data were expressed as mean ± standard deviation (SD) (*p< 0.05, **p< 0.01, vs. NC; ^∧^*p*< 0.05, ^∧∧^*p*< 0.01, vs. NC + H_2_O_2_; ^#^*p*< 0.05, ^##^*p*< 0.01, vs. TXNIP; ^‡^*p*< 0.05, ^‡‡^*p*< 0.01, vs. NC + H_2_O_2_+Gen80; ^Δ^*p*< 0.05, ^ΔΔ^*p*< 0.01, vs. NC + Gen80). HUVECs: human umbilical vein endothelial cells; TXNIP: thioredoxin-interacting protein; NC: negative control.

Expressions of senescence genes p16 and p21 in H_2_O_2_-treated HUVECs was measured by qRT-PCR and western blot. As shown in Figure 7(A–C), overexpressed TXNIP and H_2_O_2_ treatment promoted the mRNA and protein expression of p16 and p21 (*p* < 0.01), while 80 μg/mL Gen inhibited the expression of p16 and p21 (*p* < 0.01). In addition, Gen partially reversed the promotive effect of H_2_O_2_ treatment on the expression of p16 and p21 in HUVECs, while overexpressed TXNIP partially reversed the suppressive effect of Gen (Figure 7(A–C), *p* < 0.05).

**Figure 8.**
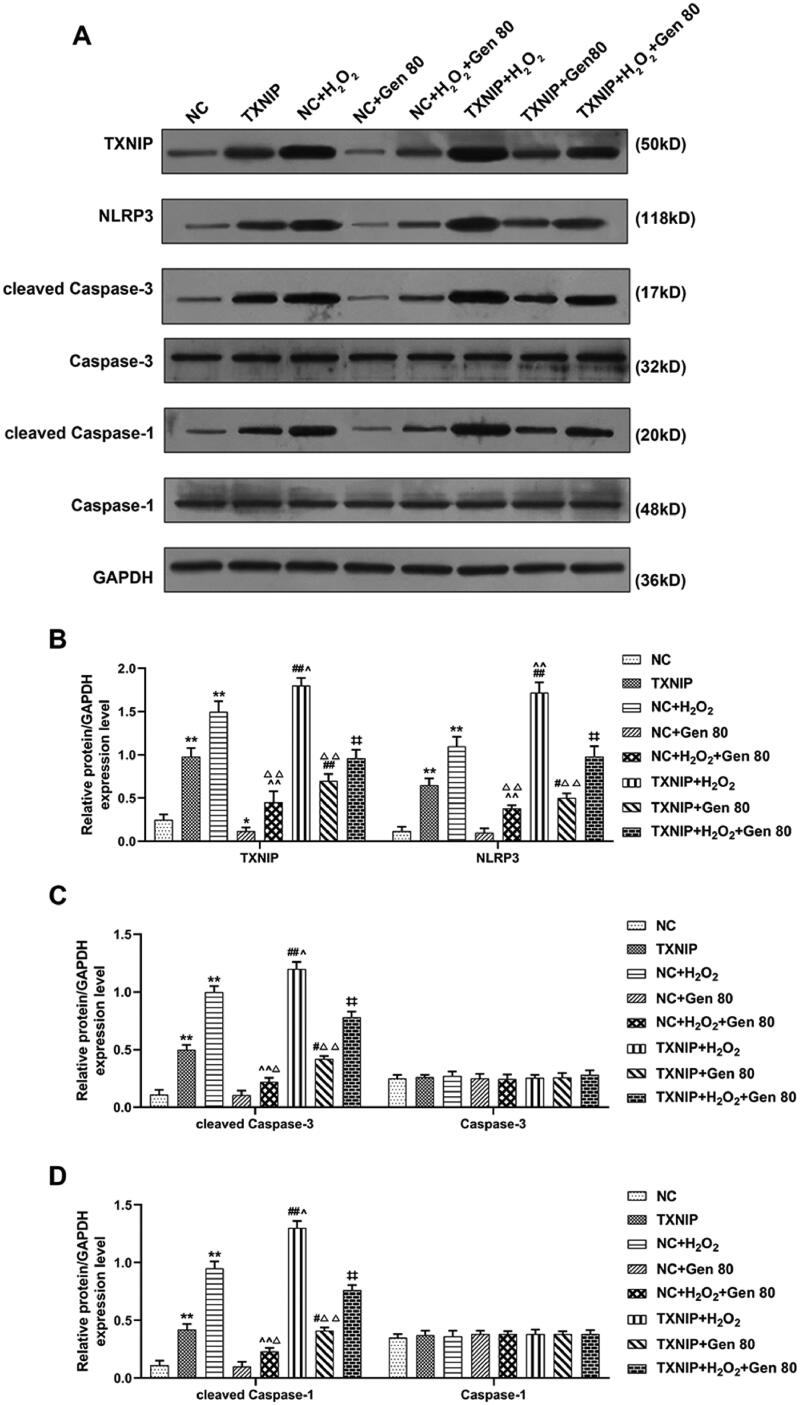
Overexpressed TXNIP partially reversed the effect of Gen on the expression of senescence-related proteins in H_2_O_2_-treated HUVECs. HUVECs were transfected with overexpressed TXNIP plasmid, treated with H_2_O_2_ or Gen alone or combination. (A–D) After transfection of overexpressed TXNIP plasmid, the expression of TXNIP, NLRP3, caspase-3, caspase-1, cleaved caspase-3, and cleaved caspase-1 in H_2_O_2_-treated HUVECs under 80 μg/mL Gen treatment was detected by western blot. GAPDH was used as the internal control. All experiments was performed in triplicate and the experimental data was expressed as mean ± standard deviation (SD) (*p< 0.05, **p< 0.01, vs. NC; ^∧^*p*< 0.05, ^∧∧^*p*< 0.01, vs. NC + H_2_O_2_; ^#^*p*< 0.05, ^##^*p*< 0.01, vs. TXNIP; ^‡‡^*p*< 0.01, vs. NC + H_2_O_2_+Gen80; ^Δ^*p*< 0.05, ^ΔΔ^*p*< 0.01, vs. NC + Gen80). HUVECs: human umbilical vein endothelial cells; TXNIP: thioredoxin-interacting protein; NC: negative control.

At the same time, the expression of senescence-related proteins TXNIP, NLRP3, cleaved caspase-3 and cleaved caspase-1 was measured by western blot. As shown in Figure 8(A–D), overexpressed TXNIP and H_2_O_2_ treatment promoted the protein expression of TXNIP, NLRP3, cleaved caspase-3 and cleaved caspase-1, while 80 μg/mL Gen inhibited the expression of TXNIP (*p* < 0.05). Moreover, Gen partially reversed the promotive effect of H_2_O_2_ treatment on the expression of TXNIP, NLRP3, cleaved caspase-3 and cleaved caspase-1 in HUVECs, while overexpressed TXNIP partially reversed the suppressive effect of Gen (Figure 8(A–D), *p* < 0.01). These findings identified that the suppressive effect of Gen on the expression of senescence genes and senescence-related proteins in H_2_O_2_-treated HUVECs was partially reversed by overexpressed TXNIP.

### Silencing of TXNIP enhanced the effect of Gen on the senescence and senescence-related proteins in H_2_O_2_-treated HUVECs

**Figure 9.**
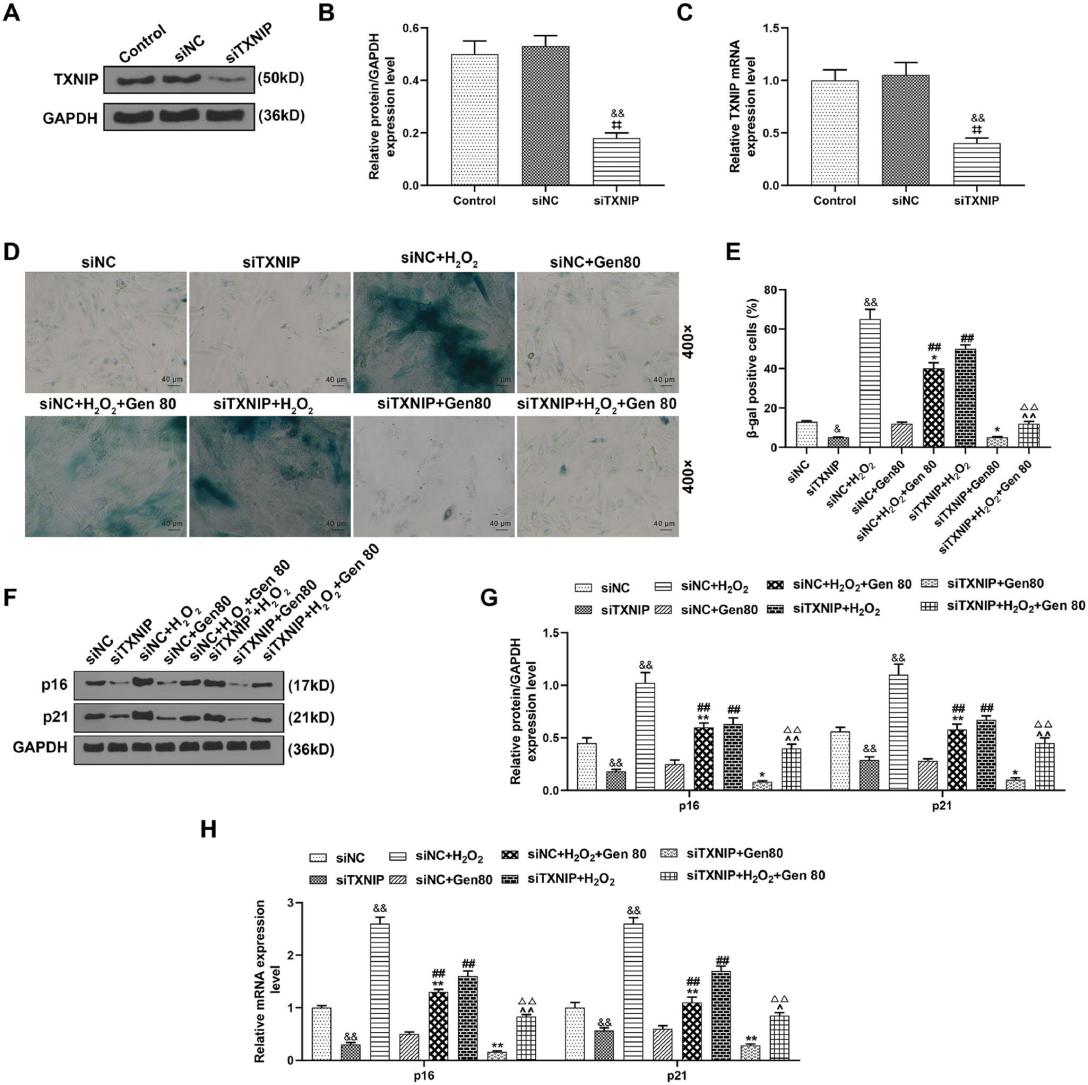
Silencing of TXNIP enhanced the effect of Gen on the senescence and senescence-related proteins expression in H_2_O_2_-treated HUVECs. (A–C) After transfection of siTXNIP, the expression of TXNIP was detected by qRT-PCR and western blot. (D, E) After transfection of siTXNIP, H_2_O_2_-induced HUVEC senescence after 80 μg/mL Gen treatment was detected by β-galactosidase staining. (F–H) After transfection of siTXNIP, the relative mRNA expression of p16 and p21 in H_2_O_2_-treated HUVECs under 80 μg/mL Gen treatment was determined by qRT-PCR and western blot.

After HUVECs transfected with siTXNIP, the expression of TXNIP was decreased (Figure 9(A–C)). It can be noted from Figure 9(D,E), the results of β-galactosidase staining identified that the percentage of β-galactosidase positive cells in siTXNIP group was lower than that in siNC group, while that in siNC + H_2_O_2_ group was higher than the siNC group (*p* < 0.05). Besides, the percentage of β-galactosidase positive cells was decreased in siTXNIP + H_2_O_2_+Gen80 group (*p* < 0.05) when compared with that in siTXNIP + H_2_O_2_ group (*p* < 0.05) and siNC + H_2_O_2_+Gen80 group, while it was increased in siNC + H_2_O_2_+Gen80 group in comparison with that in siNC + Gen80 group (Figure 9(D,E), *p* < 0.05). Furthermore, siTXNIP inhibited the expression of p16 and p21, reversed the promotive effect of H_2_O_2_ on the p16 and p21 expression, but enhanced the inhibitory effect of Gen80 on the p16 and p21 expression (*p* < 0.05, Figure 9(F–H)).

## Discussion

Endothelial cells are fragile when senescence and age-related vascular diseases occur frequently (Guo et al. 2016). Therefore, inhibiting vascular ageing may be a common target for postponing age-related vascular diseases and it is in urgent need to discover small molecules or medicine that can be used clinically to modulate endothelial cell senescence.

Senescence is an irreversible form of long-term cell-cycle arrest, caused by excessive intracellular or extracellular stress or damage (Noren Hooten and Evans 2017; Dodig et al. 2019), and cellular senescence refers to the arrest in the G1 phase of the cell-cycle (Vicencio et al. 2008). In addition, cellular senescence has been reported to be a potent anti-cancer mechanism that arrests the proliferation of mitotically competent cells to prevent malignant transformation (Chinta et al. 2015). Researchers have identified many stressors that are able to induce senescence, such as H_2_O_2_ (Song et al. 2014). Li et al. (2016) proposed that H_2_O_2_ treatment significantly inhibited the migration and proliferation of HUVECs in a dose-dependent manner. In our study, in order to establish a primary cell-induced disease model that was more clinically and physiologically relevant to human disease, H_2_O_2_-treated HUVECs were employed to discover the possible small molecules or the underlying mechanism for improving H_2_O_2_-induced senescence of HUVECs. Besides, it was noted that H_2_O_2_ inhibited the proliferation and cell cycle G1/S transition and promoted the senescence of HUVECs.

Genistein is a major isoflavone in soy with a high concentration of phytoestrogens (Amiri Gheshlaghi et al. 2017). Despite the role as a dietary supplement, Gen has been noticed because of its promising beneficial effects on various biological actions, such as cancer and inflammation (Banerjee et al. 2008). According to previous studies, Gen exerted preventive effects on prostate cancer, including inhibition of angiogenesis, inhibition of cell proliferation by inducing cell cycle arrest, and induction of apoptosis (Bilir et al. 2017). Regarding the effects of Gen on inflammation, it has been reported that Gen was able to regulate oestrogen receptor-α and oestrogen receptor-β, and suppress the progress of inflammation and angiogenesis in the murine model of peritoneal endometriosis (Sutrisno et al. 2018). Moreover, previous studies have confirmed that Gen could enhance autophagic flux and alleviate senescence in oxidized low-density lipoprotein-injured HUVECs via regulating the SIRT1/LKB1/AMPK pathway (Zhang H et al. 2019). However, whether Gen had the similar effects on H_2_O_2_-induced senescence of HUVECs still needed further investigation. Hence, in the present study, HUVECs were treated with different concentrations of Gen for 24 h. Our experimental data revealed that 20–80 μg/mL Gen had no cytotoxicity on HUVECs. Therefore, we chose 40 and 80 μg/mL Gen in the following experiments to discover the effect of Gen on H_2_O_2_-induced senescence of HUVECs. As expected, our findings proved that H_2_O_2_-induced senescence of HUVECs was mitigated by Gen.

Thioredoxin-interacting protein is a member of α-arrestin family, which functions as a multifunctional adaptor protein in various signalling pathways (Patwari et al. 2009). TXNIP plays a vital role in the negative regulation of thioredoxin (TRX) function through suppressing the reducing capacity of TRX and enhancing cellular oxidative stress (Junn et al. 2000). The suppression of TRX by TXNIP leads to cell death and promotes destructive inflammation (Spindel et al. 2012). Meanwhile, TXNIP directly activates caspase-1, cleaved caspase-1, cleaved pro-interleukin (IL)-1β and pro-IL-18, and an increase of TXNIP in young cells results in typical signs of senescence (Zhuo et al. 2010; Yang et al. 2019). The NLRP3 inflammasome belongs to nucleotide-binding and oligomerization domain-like receptors (NLRs) family (Eigenbrod and Dalpke 2015). NLRP3, adapter protein ASC and procaspase-1 are the component proteins of NLRP3 inflammasome, among which the interactions tightly regulate inflammasome function to guarantee appropriate immune activity (Ito et al. 2015). Importantly, TXNIP is identified as a binding partner of NLRP3 inflammasome, the combination of which results in the senescence of vascular endothelial cells (Yin et al. 2017). For example, Dong et al. (2020) discovered that the Wnt/β catenin pathway could be regulated by miR-20b via the TXNIP/NLRP3 axis to restrain the senescence of HUVECs. In the current study, the expression of TXNIP and NLRP3 in HUVECs were up-regulated after H_2_O_2_ treatment, which was consistent with the findings of the previous study (Tang et al. 2018). In addition, we for the first time found that the effect of H_2_O_2_ on the expression of TXNIP and NLRP3 was suppressed by Gen.

In the present study, the overexpressed TXNIP plasmid was transfected into HUVECs treated with H_2_O_2_ and Gen. Based on the experimental data, we discovered that overexpressed TXNIP partially reversed the suppressive effect of Gen on H_2_O_2_-induced senescence and apoptosis of HUVECs. Meanwhile, the expression of p16 and p21, known as cellular senescence markers (Kim et al. 2017), were promoted by overexpressed TXNIP, which partially reversed the suppressive effect of Gen on them. Caspase-3 is best known as an executioner of apoptotic cell death (Shen et al. 2017). Besides, the inhibition of Gen on the expression of cleaved caspase-3, cleaved caspase-1, TXNIP and NLRP3 in H_2_O_2_-treated HUVECs was also partially reversed by overexpressed TXNIP.

H_2_O_2_ is closely related to oxidative stress injury (Wu et al. 2018). However, the current paper inadequately discusses the effects of H_2_O_2_ on HUVECs with oxidative stress injury, and whether Gen possesses a potential protective effect on HUVECs with oxidative stress still needs further investigation. In addition, the effect of H_2_O_2_ and Gen treatments on the Ca^2+^ influx of HUVECs needs further investigation.

## Conclusions

In brief, the discoveries in the present study revealed that H_2_O_2_-induced proliferation, senescence and cell cycle G1/S transition of HUVECs were alleviated by Gen via suppressing the TXNIP/NLRP3 axis. These findings may offer a potential therapeutic approach for improving HUVEC senescence.
